# Free L-Lysine and Its Methyl Ester React with Glyoxal and Methylglyoxal in Phosphate Buffer (100 mM, pH 7.4) to Form *N*^ε^-Carboxymethyl-Lysine, *N*^ε^-Carboxyethyl-Lysine and *N*^ε^-Hydroxymethyl-Lysine

**DOI:** 10.3390/ijms23073446

**Published:** 2022-03-22

**Authors:** Svetlana Baskal, Dimitrios Tsikas

**Affiliations:** Core Unit Proteomics, Institute of Toxicology, Hannover Medical School, 30623 Hannover, Germany; baskal.svetlana@mh-hannover.de

**Keywords:** AGEs, amino acids, buffer, dicarbonyls, disproportionation, GC-MS, glycation, hydride transfer, post-translational modification (PTM), quantification

## Abstract

Glyoxal (GO) and methylglyoxal (MGO) are highly reactive species formed in carbohydrate metabolism. *N^ε^*-Carboxymethyllysine (CML) and *N*^ε^-carboxyethyllysine (CEL) are considered to be the advanced glycation end-products (AGEs) of L-lysine (Lys) with GO and MGO, respectively. Here, we investigated the reaction of free L-lysine (Lys) with GO and MGO in phosphate buffer (pH 7.4) at 37 °C and 80 °C in detail in the absence of any other chemicals which are widely used to reduce Schiff bases. The concentrations of Lys, GO and MGO used in the experiments were 0.5, 2.5, 5.0, 7.5 and 10 mM. The reaction time ranged between 0 and 240 min. Experiments were performed in triplicate. The concentrations of remaining Lys and of CML and CEL formed in the reaction mixtures were measured by stable-isotope dilution gas chromatography-mass spectrometry (GC-MS). Our experiments showed that CML and CEL were formed at higher concentrations at 80 °C compared to 37 °C. CML was found to be the major reaction product. In mixtures of GO and MGO, MGO inhibited the formation of CML from Lys (5 mM) in a concentration-dependent manner. The highest CML concentration was about 300 µM corresponding to a reaction yield of 6% with respect to Lys. An addition of Lys to GO, MGO and their mixtures resulted in strong reversible decreases in the Lys concentration up to 50%. It is assumed that free Lys reacts rapidly with GO and MGO to form many not yet identified reaction products. Reaction mixtures of Lys and MGO were stronger colored than those of Lys and GO, notably at 80 °C, indicating higher reactivity of MGO towards Lys that leads to polymeric colored MGO species. We have a strong indication of the formation of *N*^ε^-(hydroxymethyl)-lysine (HML) as a novel reaction product of Lys methyl ester with MGO. A mechanism is proposed for the formation of HML from Lys and MGO. This mechanism may explain why Lys and GO do not react to form a related product. Preliminary analyses show that HML is formed at higher concentrations than CEL from Lys methyl ester and MGO. No Schiff bases or their hydroxylic precursors were identified as reaction products. In their reactions with Lys, GO and MGO are likely to act both as chemical oxidants on the terminal aldehyde group to a carboxylic group (i.e., R-CHO to R-COOH) and as chemical reductors on labile Schiff bases (R-CH=N-R to R-CH_2_-NH-R) presumably via disproportionation and hydride transfer. Our study shows that free non-proteinic Lys reacts with GO and MGO to form CML, CEL and HML in very low yield. Whether proteinic Lys also reacts with MGO to form HML residues in proteins remains to be investigated. The physiological occurrence and concentration of HML in biological fluids and tissues and its relation to CML and CEL are elusive and warrant further investigations in health and disease. Chemical synthesis and structural characterization of HML are expected to advance and accelerate the scientific research in this topic.

## 1. Introduction

Proteinic L-lysine (Lys) residues undergo multiple post-translational modifications (PTM) including glycation of the ε-amine (*N*^ε^) group of Lys with various species. The advanced glycation end-products (AGEs) of these reactions are *N*^ε^-(1-carboxymethyl)-L-lysine (CML) and *N*^ε^-(1-carboxyethyl)-L-lysine (CEL). CML and CEL belong to the best-investigated AGEs. AGEs are widely used as markers for carbohydrate metabolism [1]. The concentration of circulating and excretory CML and CEL serves as a measure for the extent of the glycation of Lys residues in generally unknown proteins. In healthy humans, free glyoxal (GO) and methylglyoxal (MGO) occur in blood in the mid-to-upper nM-range [2,3,4] and are excreted in the urine in the very low range of 0.1 to 5.6 pmol/mmol creatinine [3,5]. Glucose as an origin of MGO is questionable [3]. The circulating and excretory concentrations of CML and CEL in healthy humans are of the order of 2 µM, and their creatinine-corrected excretion rate is of the order of 2 µmol/mmol creatinine [6]. Thus, the concentrations of MGO and GO in human biological fluids are considerably lower than those of CEL and CML. Based on the concentration of CML and CEL rather than of GO and MGO, *N*^ε^-glycation of proteins is considered to play a significant role in various diseases, notably including diabetes and cardiovascular diseases [7,8].

Over the past five decades, the formation of CML, CEL and of other Lys, arginine (Arg) and cysteine (Cys) derived AGEs and their precursors in enzymes, peptides and proteins including human and bovine albumin were often investigated using various glycating species such as reducing sugars and their glycolytic products GO and MGO. Free Lys, Arg and Cys are relatively abundant amino acids. Yet, unlike with proteins, the reactions of MGO and GO with soluble Lys and other amino acids are much less often investigated, presumably because of the general thought that only proteins are glycated. Pioneering work found that MGO was polymerized in the presence of Lys [9,10]. Moreover, MGO and GO were found to react with many amino acids in aqueous buffers in part with remarkable differences [11]. Lys was among the amino acids, which react with MGO and GO slowly, with MGO being more reactive than GO [11]. Yet, in that study, neither CML nor CEL were measured. Being highly reactive, aldehydes, MGO, GO and other carbonylic compounds may react with all of their functionalities of soluble amino acids in aqueous solutions [11]. Arg and Cys were found to be much more reactive than Lys, suggesting that not only the nucleophilic strength, for instance of the SH group of Cys, but also other structural features, for instance the formation of cyclic compounds with the guanidine group of Arg, may influence their reactions with MGO and GO.

**Figure 1:**
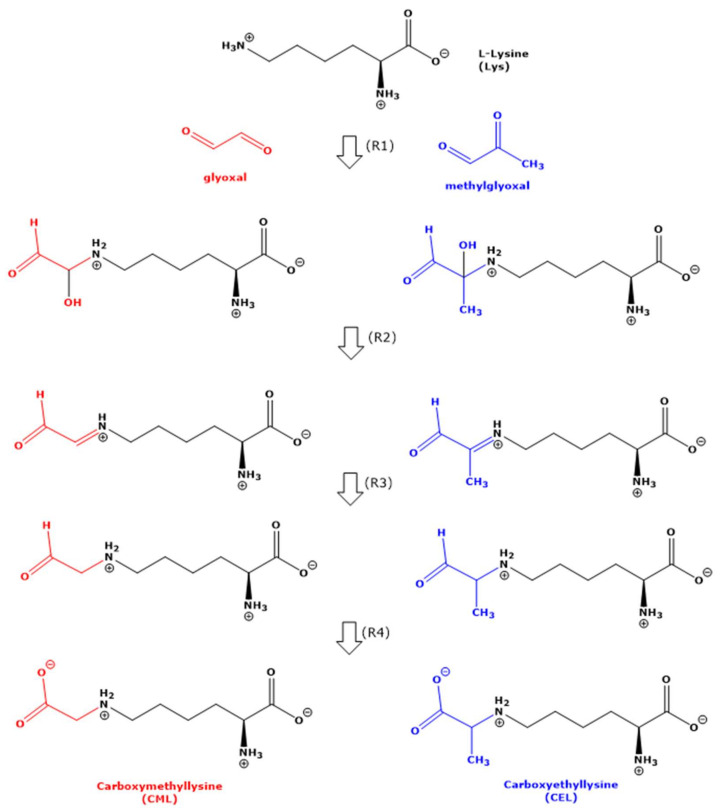
Simplified schematic of the generally assumed reactions (R1 to R4) of the *N*-terminal *N*-epsilon (*N*^ε^) amine group of free non-proteinic L-lysine (L) with glyoxal (GO) and methylglyoxal (MGO) in 100 mM phosphate-buffered (pH 7.4) at 37 °C and 80 °C finally to form carboxymethyllysine (CML) and carboxyethyllysine (CEL), respectively. For simplicity, the reaction of L-lysine with the aldehydic group of MGO is not drawn. For more details, see the text.

The first reaction product of aldehydes such as MGO and GO with amine groups including those of amino acids is considered to be a hydroxyl group-containing intermediate (i.e., -CH(OH)-NH-), which subsequently loses a molecule of water to form a Schiff base (-CH=N-) [12] (Figure 1). Schiff bases are assumed to undergo additional reactions and rearrangements finally to deliver low-molecular-mass reaction products, i.e., CML in the case of Lys with GO, and CEL in the case of Lys with MGO (Figure 1). CML and CEL are not Schiff bases but contain -CH_2_-NH- and -CH(CH_3_)-NH- as terminal groups, respectively. In vitro, the imine group of Schiff bases is chemically reduced using reagents such as NaBH_4_ [13]. In vivo, the reduction in the imine group of Schiff bases are likely to be reduced by enzymes that use NAD(P)H as the electron donor. Finally, the terminal aldehyde group needs to be oxidized to form the terminal carboxylic group of the amino acid (Figure 1). The formation of Schiff bases from the reaction of Lys with MGO is not always formulated and discussed [4].

In the present study, we investigated the reaction of free, non-proteinic Lys with GO alone, with MGO alone and with mixtures of GO and MGO in phosphate buffer of neutral pH at two different conditions. A temperature of 37 °C was used to simulate in vivo conditions, and the temperature of 80 °C was used to simulate in vitro conditions, which are often in the context of nutrients [12]. In our study, we used a recently reported gas chromatographic-mass spectrometric (GC-MS) method to study these reactions and to determine the concentration of remaining Lys and the concentrations of CML and CEL. GC-MS was also used to detect potential formation of intermediates including alcohols and Schiff bases of Lys and GO or MGO. Reactions involving GO and MGO were investigated using spectroscopic methods [14,15,16]. In the present study, we also used spectrophotometry in a few kinetic investigations.

## 2. Results

### 2.1. Kinetic Studies on the Reaction of Lys with GO and MGO to Form CML and CEL

**Figure 2:**
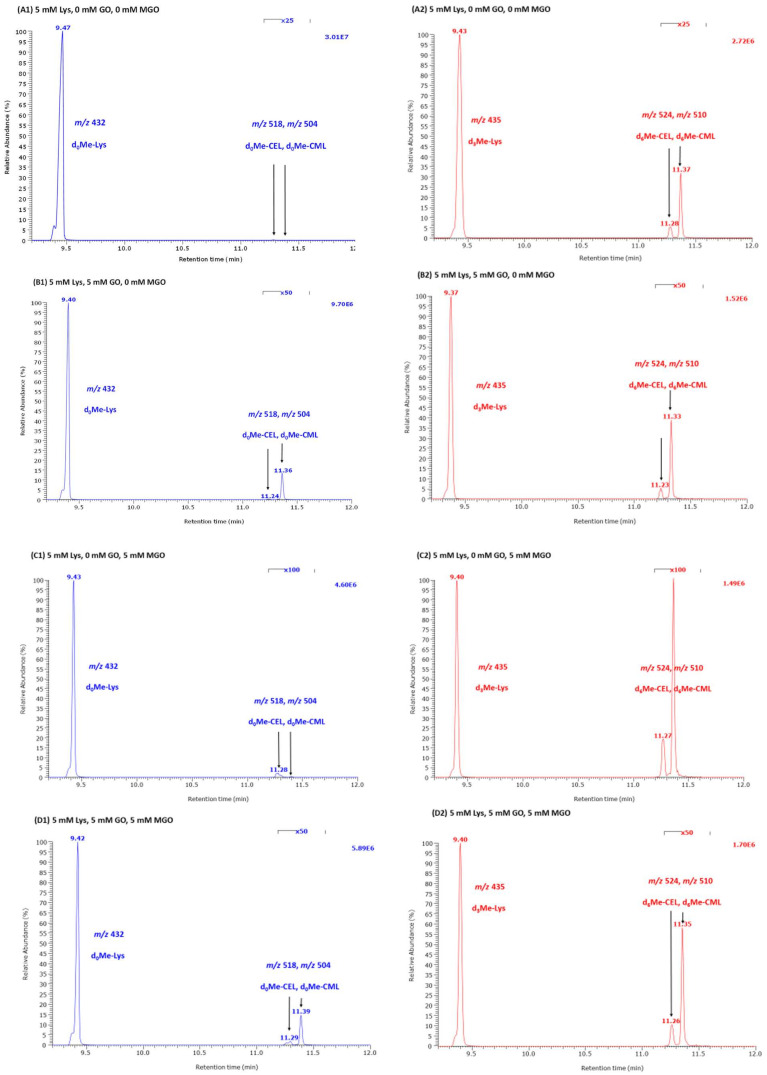
Representative partial GC-MS chromatograms from the reaction mixtures of Lys with GO, MGO and their mixture in 100 mM phosphate buffer, pH 7.4, for 1 min at room temperature. (**A**) Lys (5 mM) without GO or MGO. (**B**) Lys (5 mM) with GO (5 mM). (**C**) Lys (5 mM) with MGO (5 mM). (**D**) Lys (5 mM) with GO (5 mM) and MGO (5 mM). Selected-ion monitoring of *m*/*z* 432 for Lys, *m*/*z* for 504 for CML, *m*/*z* 518 for CEL and *m*/*z* 435, *m*/*z* 510 and *m*/*z* 524 for the respective deuterium-labeled analogs used as internal standard. The concentration of the internal standards was 500 µM for d_3_Me-Lys, 10 µM for d_6_Me-CEL and 10 µM for d_6_Me-CML. Note the magnification of the chromatogram from about 11.2 to 11.6 min. Arrows indicate the peaks of CEL and CML.

We investigated the reaction of Lys with a fixed concentration of GO (5 mM), MGO (5 mM) or their equimolar mixture (each 5 mM) at various initial concentrations (range, 0.5–10.0 mM) for varying reaction times (0 min, 1 min, 10 min, 30 min, 60 min, 240 min) at 37 °C and 80 °C. Lys, CML and CEL were measured simultaneously in the respective samples. As the reactions were not stopped, it is expected that the reaction time was each a few seconds longer than the nominal time. This especially applies to the samples 0 min and 1 min. The results of these studies are summarized in Table 1, Table 2, Table 3, Table 4, Table 5, Table 6 and Table 7. Representative GC-MS chromatograms are shown in Figure 2.

### 2.2. Remaining Lys

**Figure 3:**
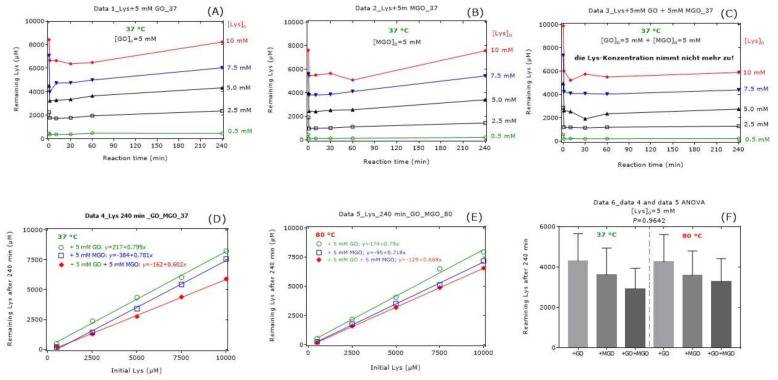
Remaining Lys concentration in reaction mixtures of Lys, GO and MGO incubated for 37 °C and 80 °C. All experiments were performed in independent triplicates. For the sake simplicity, only the mean values were plotted in (**A**–**E**). In (**F**), data are presented as mean with standard error of the mean. Linear regression analysis was performed between remaining Lys concentration (*y*) and initial Lys concentration (*x*). See also Table 1, Table 2, Table 3, Table 4, Table 5, Table 6 and Table 7.

The results of this experiment for remaining Lys are shown in Figure 3. An addition of Lys to the buffer that contained GO, MGO or GO plus MGO resulted in abrupt and comparable decreases in its concentration suggesting a reaction of Lys with GO (Figure 3A) and MGO (Figure 3B). In the presence of GO or MGO, the Lys concentration in the reaction mixtures increased after about 60 min again. Yet, in the samples containing GO and MGO, the remaining Lys did not increase from 1 min to 240 min (Figure 3C). Both at 37 °C and at 80 °C, the differences between GO and MGO were small (Figure 3D,E). The remaining Lys concentration at 240 min was borderline lower in the reaction mixture of GO compared to the reaction mixture of MGO at both temperatures (Figure 3D,E). MGO seemed to decrease the Lys concentration to a higher extent than GO at 80 °C compared to 37 °C. At an initial Lys concentration of 5 mM, there were no statistically significant differences with regard to the remaining Lys concentration at 240 min (Figure 3F). On average, the remaining Lys concentration after 240 min of reaction increased linearly with an increasing initial Lys concentration and ranged between 60% and 80%.

### 2.3. Formation of CML and CEL from Lys

The results of these experiments are summarized in Table 1, Table 2, Table 3, Table 4, Table 5, Table 6 and Table 7 and Figure 3. In the presence of MGO at an initial concentration of 5 mM and Lys (0.5–10.0 mM), CML was measured in the range 0 to 0.3 µM at 37 °C and 0 to 1.1 µM at 80 °C (Table 1, Table 2 and Table 3). In the presence of GO at an initial concentration of 5 mM and Lys (0.5–10.0 mM), CEL was not detectable at either reaction temperatures (Table 1, Table 2 and Table 3). In the presence of MGO (5 mM), the formation of CEL was dependent on the reaction time and the initial Lys concentration at both temperatures, with CEL formation being higher at 80 °C compared to 37 °C. The concentration of CEL formed after 240 min ranged from 0.6 µM to 7.4 µM at 37 °C, and from 2.5 µM to 39.0 µM at 80 °C. In the presence of GO (5 mM), the formation of CML was dependent on reaction time and initial Lys concentration at both temperatures, with CML formation being higher at 80 °C compared to 37 °C. The concentration of CML formed after 240 min ranged from 1.3 µM to 48.5 µM at 37 °C, and from 9.3 µM to 227 µM. These results indicate that CML is mainly formed from GO and Lys, while CEL is exclusively formed from MGO and Lys. The small concentrations of CML found in reaction mixtures of GO and Lys could be due to the presence of small amounts of MGO in the GO preparation or from MGO decomposed to GO during the reaction. At a molar basis, CML is formed at a higher extent from GO and Lys than CEL from MGO and Lys. The highest reaction yield of CML was 0.97% at 37 °C and 4.5% at 80 °C. The highest reaction yield of CEL was 0.1% at 37 °C and 0.8% at 80 °C.

In the presence of Lys at an initial fixed concentration of 5 mM and various MGO initial concentrations (0.5–10.0 mM), CML was measured in the range 0 to 0.4 µM at 37 °C and 0 to 0.8 µM at 80 °C (Table 4, Table 5, Table 6 and Table 7). In the presence of Lys at an initial fixed concentration of 5 mM and various GO initial concentrations (0.5–10.0 mM), CEL was not found at either reaction temperature (Table 4, Table 5, Table 6 and Table 7). In the presence of MGO (0.5–10 mM) and Lys at an initial fixed concentration of 5 mM, the formation of CEL was dependent on reaction time and temperature, with CEL formation being higher at 80 °C compared to 37 °C. The concentration of CEL formed after 240 min ranged from 0.9 µM to 5.0 µM at 37 °C, and from 2 µM to 3 µM (Table 4, Table 5, Table 6 and Table 7). In the presence of GO (0.5–10 mM) and Lys at an initial fixed concentration of 5 mM, the formation of CML was dependent on reaction time and temperature, with CML formation being higher at 80 °C compared to 37 °C. The concentration of CML formed after 240 min ranged from 2 µM to 37 µM at 37 °C, and from 7 µM to 294 µM at 80 °C (Table 4, Table 5, Table 6 and Table 7). At a molar basis, CML is formed at a higher extent from GO and Lys than CEL from MGO and Lys. The highest reaction yield of CML was 0.7% at 37 °C and 6% at 80 °C. The highest reaction yield of CEL was 0.1% at 37 °C and 0.6% at 80 °C. These results indicate that the yield of CML and CEL from Lys and GO or MGO is largely the same at 37 °C and 80 °C.

**Figure 4:**
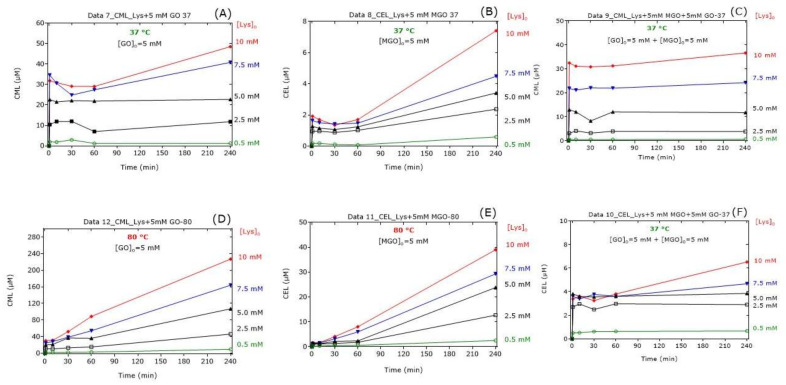
(**A**–**F**) Time-dependent formation of CML and CEL in reaction mixtures of Lys at the indicated concentrations of GO alone (5 mM), of MGO alone (5 mM) and of GO together with MGO (each at 5 mM) incubated at 37 °C or 80 °C. All experiments were performed in independent triplicates. For the sake simplicity, only the mean values are plotted. See also Table 1, Table 2, Table 3, Table 4, Table 5, Table 6 and Table 7.

In the Lys-containing reaction mixtures, GO or MGO resulted in sudden increases in the CML and CEL concentrations, respectively, in dependence on the initial Lys concentration (Figure 4). At 37 °C, the CML and CEL concentrations were relatively constant for the first 60 min and increased thereafter, with the CEL concentrations increasing more strongly. At 80 °C, the CML and CEL concentrations increased almost linearly with reaction time to reach higher concentrations compared to 37 °C. In the Lys-, GO- and MGO-containing reaction mixtures, the addition of Lys resulted in sudden increases in the CML (Figure 4C) and CEL (Figure 4F) concentrations in dependence on the initial Lys concentration, yet almost independent of the reaction time. In the co-presence of GO and MGO, the formation of CML (Figure 4C) and CEL (Figure 4F) from Lys was almost independent of the reaction time after the first minute of the reaction.

Figure 3 and Figure 4, Table 1, Table 2, Table 3, Table 4, Table 5, Table 6 and Table 7 indicate that the loss of Lys is much higher than the formation of CML and CEL, suggesting loss of Lys by additional not investigated reactions.

### 2.4. Formation of CML, CEL and HML from Lys Methyl Ester

We performed separate reactions of MGO (5.8 mM) and GO (8.8 mM) with unlabeled methyl ester of Lys (d_0_Me-Lys, 1 mM) in the absence and in the presence of hydrogen peroxide (H_2_O_2_, 5 mM). After reaction for 1 h, the reaction mixtures (*n* = 6) were halved. One series of samples (a) was derivatized with 2 M HCl in CH_3_OH; the second series of samples (b) was derivatized with 2 M HCl in CD_3_OD to generate the trideuteromethyl ester of Lys (d_3_Me-Lys). Then, both samples were derivatized separately with pentafluoropropionic anhydride (PFPA) and analyzed by GC-MS for unlabeled and deuterium-labeled Lys, CML and CEL in the SIM mode (*m*/*z* 432, 435, 504, 510, 518, 524).

In this experiment, we found d_0_Me-Lys and d_3_Me-Lys in all reaction mixtures. The ratio of the peak area of d_3_Me-Lys in sample (a) and sample (b) ranged between 0.0025 and 0.0041 in all reaction mixtures (*n* = 6). The ratio of the peak area of d_0_Me-Lys in sample (a) and sample (b) ranged between 2.13 and to 3.78 in all reaction mixtures (*n* = 6), indicating some hydrolysis of d_0_Me-Lys and d_3_Me-Lys during the reaction and/or derivatization. In confirmation of previous results, CML was detected in the d_0_Me-Lys+GO samples, whereas CEL was detected only in the d_0_Me-Lys + MGO samples. The presence of H_2_O_2_ did not influence remarkably the formation of unlabeled deuterium-labeled CML or CEL (data not shown). The derivatized samples that did not contain H_2_O_2_ were analyzed in quintuplicate after 6 days of storage at room temperature. The 1000-fold higher peak area ratio values were (mean ± SD) 3.7 ± 0.57 (RSD, 15%) for d_0_Me-CML-to-d_0_Me-Lys and 2.1 ± 0.27 (RSD, 13%) for d_3_Me-CML-to-d_3_Me-Lys in mixtures of Lys and GO, and 0.37 ± 0.02 (RSD, 6.4%) for d_0_Me-CEL-to-d_0_Me-Lys, and 0.27 ± 0.01 (RSD, 3.5%) for d_3_Me-CEL-to-d_3_Me-Lys in mixtures of Lys and MGO, indicating a higher yield of CML compared to CEL. The corresponding values were 1.07 ± 0.09 (RSD, 9%) for d_0_Me-Lys-to-d_3_Me-Lys in GO-containing samples and 1.18 ± 0.16 (RSD, 14%) for d_0_Me-Lys-to-d_3_Me-Lys in MGO-containing samples.

**Figure 5:**
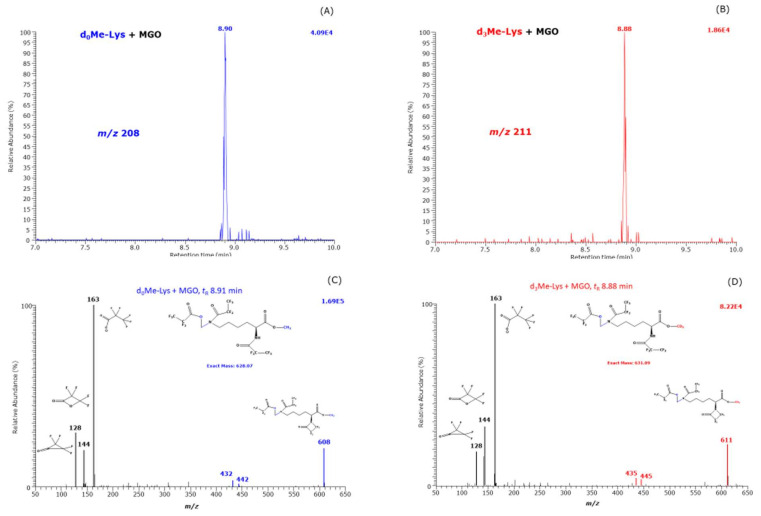
Extracted ion chromatograms of *m*/*z* 608 (**A**) and *m*/*z* 611 (**B**) and negative-ion chemical ionization mass spectra (**C**) and (**D**) in the *m*/*z* range 50–650 of the Me-PFP derivatives of reaction products formed from the reaction of 1 mM unlabeled methyl ester of Lys (d_0_Me-Lys) with MGO (5.8 mM) and 1 mM trideuteromethyl ester of Lys (d_3_Me-Lys) with MGO (5.8 mM) in 100 mM buffer, pH 7.4, for 1 h at 37 °C. Insets in (**C**) and (**D**) indicate the proposed structures of the mass fragments. The retention times of 8.91 min and 8.88 min are close to the retention time of the Me-PFP derivatives of synthetic d,l-5-hydroxy-lysine [6].

In addition, the reaction products were analyzed by scanning (*m*/*z* range, 50 to 650) in order to test formation of other reaction products besides CML and CEL. We observed a peak eluting at about 8.9 min only in the samples that contained MGO both in the absence and in the presence of H_2_O_2_ suggesting independency of H_2_O_2_. The extracted ion chromatograms and the mass spectra of these reaction products are shown in Figure 5. They are very similar to those of d,l-5-hydroxy-lysine [6]. The mass fragments at *m*/*z* 128, *m*/*z* 144 and *m*/*z* 163 are common to d_0_Me-Lys and d_3_Me-Lys derivatives and indicative of a hydroxy-derivative. The largest mass fragments were observed at *m*/*z* 608 and *m*/*z* 611 indicative of unlabeled and trideuteromethyl monoesters, respectively. The mass fragments at *m*/*z* 608 and *m*/*z* 611 are most likely formed from a neutral loss of 20 Da due to HF ([M−HF]^−^). Such a fragmentation is very common in *N*^ε^-pentafluoropropionyl derivatives of ε-amino acids [6]. The [M−HF]^−^ ions in the Me-PFP derivatives of d,l-5-hydroxy-lysine are *m*/*z* 594 for d_0_Me and *m*/*z* 597 for d_3_Me, i.e., by a methylene group (CH_2_, 14 Da) smaller. The retention times and the mass spectra of the Me-PFP derivatives of the new reaction suggest that this reaction product is presumably *N*^ε^-(hydroxymethyl)-lysine methyl ester. The retention times of the Me-PFP derivatives of d,l-5-hydroxy-lysine were 8.54 min and 8.66 min [6]. The slightly longer retention time of the Me-PFP derivative of 8.9 min is presumably in part due to the additional methylene group in *N*^ε^-(hydroxymethyl)-lysine methyl ester.

**Figure 6:**
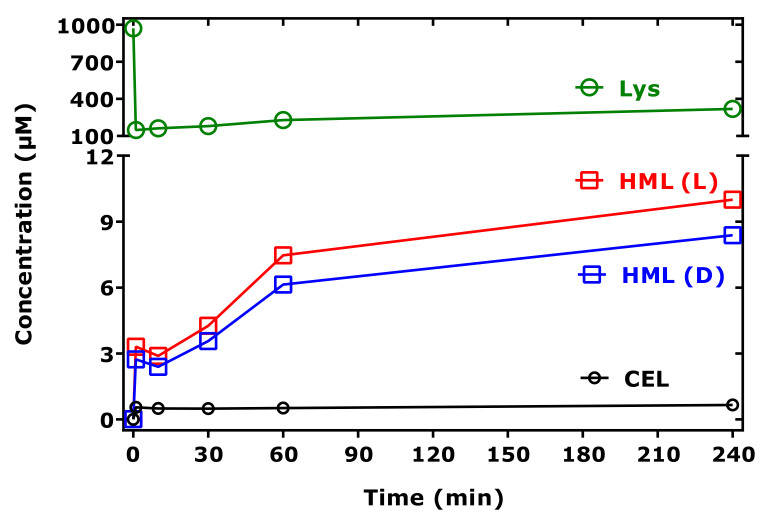
Time-course of the formation of HML and CEL and of remaining Lys in reaction mixtures of Lys methyl ester (1 mM) and MGO (5.8 mM) in 100 mM buffer, pH 7.4, at 37 °C. (L) and (D) indicate L-5-hydroxy-lysine and D-5-hydroxy-lysine (each 20 µM), which were used as the internal standards for HML. See also Figure 7.

**Figure 7:**
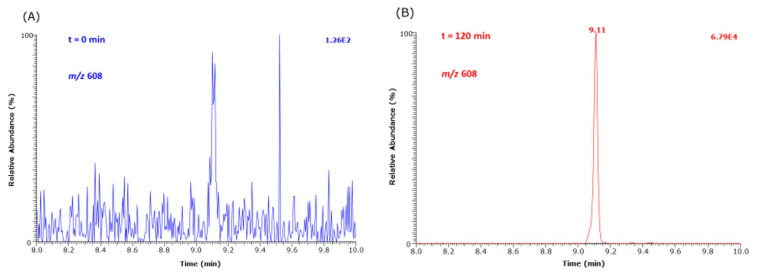
Partial GC-MS chromatograms of selected ion monitoring of *m*/*z* 608 of the Me-PFP derivative of HML formed from the reaction of 1 mM Lys methyl ester with 5.8 mM MGO at 37 °C in 100 mM buffer, pH 7.4, (**A**) before addition (t = 0 min) and (**B**) after addition (t = 120 min) of MGO.

As *N*^ε^-(hydroxymethyl)-lysine (HML) is not commercially available as a synthetic reference compound, we used d,l-5-hydroxy-lysine as an internal standard to estimate the order of formation of HML from Lys methyl ester and MGO in buffer at 37 °C. Figure 6 shows that HML is formed at higher concentrations than CEL under identical experimental conditions. Figure 7 shows partial GC-MS chromatograms before (t = 0 min) and after (t = 240 min) the addition of MGO.

### 2.5. Spectrophotometric Analyses

The experimental conditions for and the results from these analyses are reported in the Supplementary Materials of this work. After addition of the chemicals to the buffer, the maximum wavelengths were 208 nm for Lys in the absence of MGO and 273 nm for MGO in the absence of Lys (Figure S1). The ultraviolet/visible (UV/vis) spectra of mixtures containing both Lys and MGO differed from those of Lys and MGO alone and were dependent upon the order of their addition to the buffer. Higher absorbance values were obtained when first Lys and then MGO were added into the cuvette (Figure S1). The UV/vis spectra of the reaction mixtures show Lys-concentration-depending absorbance values, which were higher in the presence of MGO and GO + MGO compared to GO (Figure S2). In the presence of GO and MGO, GO only marginally reduced the absorbance values caused by Lys and MGO. The absorbance at 273 nm of the sample that contained MGO but not Lys only little changed with reaction time. The absorbance at 273 nm of the sample that contained MGO and Lys increases more strongly with reaction time (Figure S3). The time-course of the formation of CML in reaction mixtures of free Lys (1 mM) or Lys methyl ester (1 mM) GO alone (8.8 mM) in distilled water differed from that in 100 mM phosphate buffer, pH 7.4 (Figure S4).

The reaction mixtures of Lys and GO or Lys and MGO were colorless at 37 °C at all concentrations tested. At 80 °C, the reaction mixtures of Lys and GO were slightly yellowish only at high concentrations. At this temperature, the reaction mixtures of Lys and MGO were yellow at lower and brownish at higher concentrations.

## 3. Discussion

The aim of the present study was to investigate the formation of CML and CEL from free non-proteinic Lys, GO and MGO in an aqueous buffer of pH 7.4 without the use of additional chemical and enzymatic treatments which are commonly used in the research area of AGEs. Thus, in our study, we did not use reducing reagents such as NaBH_4_ to reduce intermediate Schiff bases of Lys with GO and MGO. We also did not use any enzymes to oxidize their terminal aldehyde groups to form the dicarboxylic acids CML and CEL. We used a previously reported GC-MS method [6] for the specific measurement of CML, CEL and Lys in their reaction mixtures.

The absence of the expected aldehydic Schiff bases and the formation of CML and CEL under the experimental conditions of our study suggest that disproportionation and hydride transfer are likely to have occurred in the expected Schiff bases. In the case of Lys (residue R), this could involve a reduction in OCH-CH=N-R (with GO) and in -C(CH_3_)=N-R (with MGO) to the OCH-CH_2_-NH-R and OCH-C(CH_3_)-NH-R groups, respectively, and the oxidation of their terminal OCH-groups to their carboxylic acids HOOC-CH_2_-NH-R in the case of CML and HOOC-C(CH_3_)-NH-R in the case of CEL. Such a disproportionation was described for free glyoxal to glycolic acid [14]). Formation of CEL from the reaction of Lys residues of bovine serum albumin (BSA) with MGO was reported in the absence of any reductive and oxidative treatment of reaction products [17]. To our knowledge, the requirement of reductive and oxidative treatments to convert the aldehydic Schiff bases formed by free Lys and of Lys residues in proteins with GO or GMO is not reported thus far. Yet, our study cannot exclude the formation of the expected aldehydic Schiff bases because the GC-MS method has not been developed for their analyses. The formation of a Schiff base from the reaction of Lys with MGO is rarely addressed, explicitly formulated or discussed in the literature [4].

Inorganic polyvalent anions including phosphate and carbonate were shown to catalyze the formation of MGO from glyceraldehyde and dihydroxyacetone [18]. In our study, we used sodium phosphate (100 mM, pH 7.4) to prepare the buffer. It is, therefore, possible that under the experimental conditions of our study, phosphate anions were involved in the formation of CEL and possibly of CML. Yet, we consider that possible effects of phosphate on the reaction of Lys with MGO and GO were constant.

GO and MGO exist in aqueous solutions in their free forms, as monohydrates and dehydrates. In addition, MGO and possibly GO may form polymers in the presence of Lys albeit at much higher concentrations and temperatures than those used in the present study [9,10]. In our study, we did not measure MGO and GO, but only qualitatively by spectrophotometry.

Loss of Lys in our study could also be due to the formation of ε-caprolactam from esterified Lys [19]. Lys could also react with GO and MGO via its α-amine group as well as with its carboxylic group, with the latter presumably yielding lysinate derivatives [20]. In our study, we did not analyze formation of such Lys derivatives. In some experiments, Lys concentration increased again at higher reaction times. Decomposition of unknown Lys derivatives to Lys may have resulted in such increases. CEL is less stable than CML and decomposes to Lys [6]. This may have decreased the extent of the actual yield of CEL and increased the concentration of Lys. Lys-containing samples treated with MGO become stronger brownish compared to GO presumably due to the formation of polymeric melanoids [10]. In our study, we did not further investigate this issue.

Our study revealed, for the first time, strong evidence of the formation of hydroxymethyl-lysine (HML) in reaction mixtures of Lys and MGO. First, semi-quantitative analyses using d,l-5-hydroxy-lysine as a surrogate internal standard suggest that HML is formed at higher yields than CML and CEL. HML is rarely reported in the literature, mostly in the context of formyl-lysine [21] and hydroxylation of histone-derived monomethyl-lysine [22]. Synthetic HML was found to be a source of bioavailable Lys in ruminants [23]. HML can be easily obtained by the reaction of Lys with formaldehyde in Ca(OH)_2_-containing aqueous solution [23]. MGO is considered to form spontaneously formaldehyde and acetaldehyde [10]. In our study, we did not test this possibility. Due to the presumably large number of chemically reactive species from decomposed, hydrated or polymerized MGO [24], other reactions in Lys- and MGO-containing aqueous solutions may also occur.

**Figure 8:**
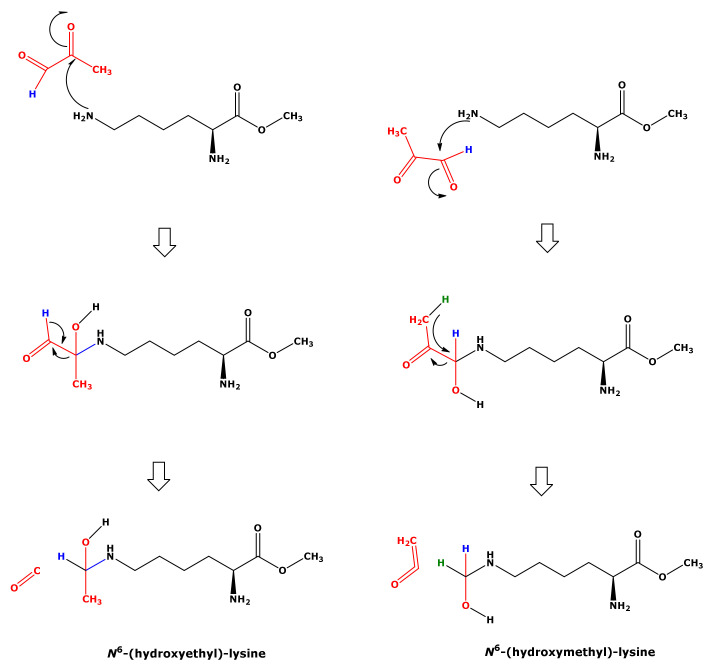
Proposed mechanism for the formation of hydroxymethyl-lysine (right panel) from the reaction of free lysine methyl ester with methylglyoxal (MGO) in 100 mM phosphate buffer (pH 7.4). This mechanism includes nucleophilic attack of the terminal amine group on the aldehyde group of MGO and intramolecular hydride transfer. Nucleophilic attack of the terminal amine group on the keto group of MGO would lead to the formation of hydroxyethyl-lysine (left panel), which however was not observed in the present study.

In our study, the reaction of Lys with MGO did not result in the formation of hydroxyethyl-lysine (HEL). We propose a mechanism for the formation of HML from Lys and MGO (Figure 8). This mechanism includes nucleophilic attack of the terminal amine group of Lys on the aldehyde group of MGO and intramolecular hydride transfer. That Lys and GO did not form HML, analogous to Lys and MGO (Figure 8), could be due to differences in the leaving groups. The nucleophilic attack of the terminal amine group of Lys on the carbonyl groups of GO and MGO is considered to form hydroxylic compounds as intermediates that subsequently form the corresponding Schiff bases (Figure 1). In our study, we did not identify any Schiff bases. Yet, this does not exclude their possible formation.

The identification of HML as a reaction product of Lys and MGO may suggest that the first reaction product between Lys and MGO is more stable than that of Lys and GO. This may be an explanation for the formation of CEL with lower yield compared to CML.

GO and MGO react with Lys residues via crosslinking in proteins to form GO-Lys dimer (GOLD) and MGO-Lys dimer (MOLD) [25]. GOLD and MOLD are permanently positively charged imidazolium compounds and not accessible to GC-MS analysis. We therefore did not analyze such species in the present study.

Our in vitro study may have some limitations. We used very high concentrations of GO and MGO (range, 0.5–10 mM), which are, however, commonly used in similar studies. We performed the experiments at two temperatures to simulate physiological conditions and those often used in the context of nutrients including milk and dairy products. In proteolysates, free Lys is found in mM-concentrations, yet the concentration of GO and MGO are expected to be very low and to lie rather in the lower µM-range. Preliminary experiments using physiologically relevant concentrations of GO and MGO did not allow demonstration of a formation of CML and CEL despite the use of a very sensitive GC-MS method. At high concentrations of Lys, GO and MGO, our study demonstrates the formation of CML, CEL and HML in 100 mM phosphate buffer (pH 7.4) without the need of additional chemicals and enzymes. Yet, the yield of CML, CEL and HML is very low. Whether CML, CEL and HML are formed at higher concentrations from reactions of GO and GMO with Lys in the presence of reductors and oxidants should be a subject of further investigations.

In summary, our study shows that, in absence of any reductors and oxidants, free Lys reacts with GO to form CML, and with MGO to form CML at a very low extent. We demonstrate for the first time that the reaction of Lys methyl ester with MGO, but not the reaction of Lys methyl ester with GO, generates HML. HML seems to be formed at higher concentrations than CML and CEL. We propose mechanisms for the formation of CML, CEL and HML, which include disproportionation and hydride transfer. These mechanisms are supported by the literature but remain to be investigated using appropriate chemicals such as deuterated GO and MGO. Chemical synthesis of HML will facilitate investigations on the physiological occurrence of HML in biological samples and its action on the receptors of AGEs (RAGEs) [26]. It is worth mentioning that CML and CEL have a very low affinity to RAGEs [26]. The hydroxy group of HML could increase its affinity to RAGEs. The significance of HML in health and disease, notably in diabetes, remains to be investigated. In case of the occurrence of HML in biological samples at higher concentrations than CML and CEL, HML could turn out to be a better biomarker of glycation.

## 4. Materials and Methods

### 4.1. Chemicals, Materials and Reagents

Glyoxal and methylglyoxal were obtained from Sigma-Aldrich (Steinheim, Germany) each as 40 wt.% solution in water. L-Lysine, *N*^ε^-(1-carboxymethyl)-L-lysine (chemical purity, 95%) and *N*^ε^-(1-carboxyethyl)-L-lysine (chemical purity, 95%) were purchased from Cayman (Ann Arbor, Michigan, USA). Tetradeuterated methanol (CD_3_OD, 99% at ^2^H) and pentafluoropropionic anhydride (PFPA) were supplied by Sigma-Aldrich (Steinheim, Germany). Methanol was obtained from Chemsolute (Renningen, Germany). Ethyl acetate (EA) was obtained from Merck (Darmstadt, Germany). Hydrochloric acid (37 wt.%) was purchased from Baker (Deventer, The Netherlands) and was used to prepare the esterification reagent (2 M HCl in CH_3_OH or 2 M HCl in CD_3_OD). Glassware for GC-MS (1.5-mL autosampler vials and 0.2-mL microvials) and the fused-silica capillary column Optima 17 (15 m × 0.25 mm I.D., 0.25-µm film thickness) were purchased from Macherey-Nagel (Düren, Germany). Spectrophotometric analyses were performed at room temperature on the spectrophotometer model Specord 50 from Analytik Jena (Jena, Germany) using 1-cm cuvettes (UVetten, Eppendorf, Hamburg, Germany). Scans were performed in the range 190–500 nm (1 s per cycle).

### 4.2. Procedure for the Reaction of Lys with GO and MGO

The kinetic studies were performed in 100 mM sodium phosphate buffer, pH 7.4, in triplicate on three different days at either 37 °C in a thermostate (model MBT250; Kleinfeld, Gehrden, Germany) or 80 °C in a drying oven (model T6; Heraeus, Hanau, Germany). Lys (100 mM in distilled water), GO and MGO (each 100 mM in phosphate buffer daily prepared) stock solutions were prepared and used to reach desired concentrations in reaction mixtures. The incubation mixtures (500 µL final volume) in Eppendorf tubes contained Lys, GO, MGO or an equimolar mixture of GO and MGO at varying concentrations (0.5, 2.5, 5, 7.5, 10 mM) as specified in the Results section. If not otherwise specified, Lys was first added to the reaction mixtures which were then left to equilibrate at 37 °C of 80 °C for 30 min. Reactions were started with the addition of GO, MGO or GO plus MGO (varying volumes in the range 2.5 to 50-µL aliquots of their 100 mM solutions). The first samples were taken immediately after the Lys addition, then transferred into autosampler glass vials, and the liquid was evaporated to dryness under a stream of nitrogen glass. The incubation time of this sample corresponds to time zero (0 min). Subsequently, each 10-µL sample was taken from the incubation mixtures at 1, 10, 30, 60 and 240 min, transferred to glass vials and evaporated therein to dryness under a stream of nitrogen.

The residues obtained by solvent evaporation were subjected to a two-step derivatization procedure as described elsewhere [6]. They were reconstructed in 100-µL aliquots of a 2 M HCl/CH_3_OH solution; the glass vials were tightly sealed and heated for 60 min at 80 °C to prepare methyl esters of amino acids. After cooling to room temperature, the samples were spiked with 10-µL aliquots of newly synthesized trideuteromethyl esters of Lys, CML and CEL using a 2 M HCl/CD_3_OD solution used as the internal standards. In quantitative analyses, the final concentrations of the internal standards were 500 µM for d_3_Me-Lys, 10 µM for d_6_Me-CEL and 10 µM for d_6_Me-CML. After cooling to room temperature and sample evaporation to dryness under a stream of nitrogen, 100-µL aliquots of a freshly prepared PFPA solution in ethyl acetate (PFPA-EA, 1:4, v/v) were added; the glass vials were tightly sealed and heated for 30 min at 65 °C to prepare *N*-pentafluoropropionic amides of the methyl esters (Me-PFP) of amino acids. After cooling to room temperature and sample evaporation to dryness under a stream of nitrogen gas, the residues were treated first with 200-µL aliquots of 400 mM borate buffer, pH 8.5, and immediately thereafter with 200-µL aliquots of toluene. The mixtures were immediately vortex-mixed for 60 s and centrifuged (4000× *g*, 5 min, 18 °C) to remove acidic components and to extract the Me-PFP derivatives of the reaction products into toluene. Finally, aliquots (150 µL) of the upper organic phase were transferred into 1.5-mL autosampler glass vials equipped with 200-µL microinserts; the samples were sealed and subjected to quantitative GC-MS analysis as described below.

### 4.3. GC-MS Conditions and Quantitative Analyses of Lys, CML, CEL and HML in Reaction Mixtures of Lys with GO and MGO

The concentration of Lys, CML, CEL was determined simultaneously by GC-MS using their respective internal standards as described elsewhere [6]. The concentration of HML was determined by GC-MS using d,l-5-hydroxy-lysine as the internal standard. Analyses were performed on a GC-MS apparatus model ISQ. Toluene aliquots (1 µL) were injected in the splitless mode. The 10-µL Hamilton needle of the autosampler was cleaned automatically three times with toluene (5 µL) after each injection. Injector temperature was kept at 280 °C. Helium was used as the carrier gas at a constant flow rate of 1.0 mL/min. The oven temperature was held at 40 °C for 0.5 min and ramped to 210 °C at a rate of 15 °C/min and then to 320 °C at a rate of 35 °C/min. Interface and ion-source temperatures were set to 300 °C and 250 °C, respectively. Electron energy was 70 eV and electron current 50 µA. Methane was used as the reagent gas for negative-ion chemical ionization at a constant flow rate of 2.4 mL/min. Quantitative analyses were performed in the selected-ion monitoring (SIM) mode at an electron multiplier voltage of 1400 V. The ions with mass-to-charge (*m*/*z*) were *m*/*z* 432 for Lys, *m*/*z* 504 for CML and *m*/*z* 518 for CEL, and *m*/*z* 435, *m*/*z* 510 and *m*/*z* 524 for the respective internal standards. The dwell time was 50 ms for each ion. In qualitative analyses, the ions with *m*/*z* 430, *m*/*z* 502 and *m*/*z* 516 were monitored for the putative Schiff bases of Lys, CML and CEL. Mass spectra of reaction products of Lys with GO or MGO were generated after their derivatization by scanning the quadrupole in the *m*/*z*-range 50 to 1000. The retention times of the internal standards observed in a series of quantitative analyses (*n* = 148) were (mean ± standard deviation (SD)) 9.406 ± 0.023 min (relative standard deviation (RSD), 0.25%) for Lys, 11.36 ± 0.022 min (0.19%) for CEL and 11.27 ± 0.022 min (0.20%) for CML. The mean relative retention time was 1.19 for d_3_Me-CEL/d_3_Me-Lys, 1.199 for d_3_Me-CML/d_3_Me-Lys and 1.001 for d_3_Me-CML/d_3_Me-CEL and varied by 8.3% each. The corresponding peak area values (arbitrary units) of the internal standards in these analyses were 4.2 × 10^6^ (RSD, 28%), 1.19 × 10^4^ (RSD, 51%) and 4.47 × 10^4^ (RSD, 46%).

### 4.4. Statistical Analysis and Data Presentation

Data analyses were performed using GraphPad Prism 7 for Windows (GraphPad Software, San Diego, CA, USA). Data are presented as mean ± SD or mean ± SEM.

## Figures and Tables

**Table 1:** Concentration (mean ± SD; µM) of remaining Lys and formed CML and CEL from the reaction of Lys and GO at initial concentrations of 500–10000 µM and 5000 µM, respectively, at 37 °C at 80 °C for the indicated times (min) in 100 mM phosphate buffer (pH 7.4). n.d., not detected.

[Lys]_o_	[GO]_o_	Time	[Lys]	[CML]	[CEL]	[Lys]	[CML]	[CEL]
			37 °C			80 °C		
500	5000	0	470 ± 51	0.003 ± 0.004	n.d.	496 ± 143	0.00 ± 0.00	n.d.
500	5000	1	366 ± 91	1.97 ± 0.59	n.d.	432 ± 101	2.05 ± 0.65	n.d.
500	5000	10	379 ± 103	1.87 ± 0.72	n.d.	422 ± 87	1.77 ± 0.97	n.d.
500	5000	30	387 ± 91	2.95 ± 1.24	n.d.	425 ± 54	2.05 ± 1.24	n.d.
500	5000	60	479 ± 58	1.20 ± 0.72	n.d.	447 ± 61	2.52 ± 2.19	n.d.
500	5000	240	470 ± 49	1.25 ± 0.81	n.d.	525 ± 31	9.29 ± 1.74	n.d.
								
2500	5000	0	2255 ± 141	0.01 ± 0.02	n.d.	2222 ± 256	0.01 ± 0.02	n.d.
2500	5000	1	1790 ± 209	10.4 ± 4.8	n.d.	1712 ± 319	10.8 ± 4.0	n.d.
2500	5000	10	1737 ± 315	11.9 ± 3.5	n.d.	1686 ± 265	10.2 ± 2.9	n.d.
2500	5000	30	1791 ± 314	12.0 ± 2.7	n.d.	1749 ± 306	12.9 ± 5.1	n.d.
2500	5000	60	1964 ± 126	7.0 ± 1.5	n.d.	1893 ± 256	15.1 ± 8.9	n.d.
2500	5000	240	2372 ± 468	11.8 ± 2.4	n.d.	2182 ± 144	46.0 ± 3.0	n.d.
								
5000	5000	0	4509 ± 332	0.03 ± 0.06	n.d.	4287 ± 342	0.02 ± 0.03	n.d.
5000	5000	1	3226 ± 518	22.6 ± 6.8	n.d.	3078 ± 698	20.1 ± 7.9	n.d.
5000	5000	10	3284 ± 570	21.5 ± 5.6	n.d.	3276 ± 622	21.1 ± 9.0	n.d.
5000	5000	30	3352 ± 401	22.1 ± 7.1	n.d.	3706 ± 272	36.6 ± 4.3	n.d.
5000	5000	60	3648 ± 356	21.9 ± 3.2	n.d.	3920 ± 176	36.0 ± 3.3	n.d.
5000	5000	240	4338 ± 742	22.7 ± 9.6	n.d.	4059 ± 245	107 ± 14	n.d.
								
7500	5000	0	7080 ± 347	0.02 ± 0.02	n.d.	6084 ± 647	0.01 ± 0.02	n.d.
7500	5000	1	3985 ± 1800	34.6 ± 6.8	n.d.	4688 ± 939	24.1 ± 13.2	n.d.
7500	5000	10	4739 ± 788	30.6 ± 8.1	n.d.	4798 ± 880	28.2 ± 6.7	n.d.
7500	5000	30	4759 ± 725	24.8 ± 11.7	n.d.	4742 ± 859	38.6 ± 15.5	n.d.
7500	5000	60	5001 ± 572	27.4 ± 3.8	n.d.	5524 ± 1945	54.0 ± 20.4	n.d.
7500	5000	240	6040 ± 290	40.8 ± 8.2	n.d.	6508 ± 916	164 ± 25	n.d.
								
10,000	5000	0	8430 ± 1396	0.02 ± 0.03	n.d.	8135 ± 768	0.01 ± 0.02	n.d.
10,000	5000	1	6656 ± 1004	31.8 ± 6.7	n.d.	6297 ± 1370	30.0 ± 10.6	n.d.
10,000	5000	10	6652 ± 1392	30.9 ± 10.2	n.d.	6340 ± 1143	31.0 ± 9.6	n.d.
10,000	5000	30	6374 ± 839	29.1 ± 6.9	n.d.	6064 ± 940	51.9 ± 27.8	n.d.
10,000	5000	60	6494 ± 675	29.0 ± 6.5	n.d.	6802 ± 1064	88.6± 42.7	n.d.
10,000	5000	240	8235 ± 954	48.5 ± 14.9	n.d.	7939 ± 634	227 ± 37	n.d.

**Table 2:** Concentration (mean ± SD; µM) of remaining Lys and formed CML and CEL from the reaction of Lys and MGO at initial concentrations of 500–10,000 µM and 5000 µM, respectively, at 37 °C at 80 °C for the indicated times (min) in 100 mM phosphate buffer (pH 7.4).

[Lys]_o_	[MGO]_o_	Time	[Lys]	[CML]	[CEL]	[Lys]	[CML]	[CEL]
			37 °C			80 °C		
500	5000	0	454 ± 62	0.00 ± 0.00	0.00 ± 0.00	443 ± 45	0.00 ± 0.00	0.00 ± 0.00
500	5000	1	105 ± 12	0.08 ± 0.02	0.18 ± 0.16	131 ± 89	0.07 ± 0.04	0.26 ± 0.03
500	5000	10	102 ± 37	0.08 ± 0.06	0.18 ± 0.15	146 ± 65	0.04 ± 0.04	0.28 ± 0.05
500	5000	30	116 ± 92	0.10 ± 0.05	0.11 ± 0.16	118 ± 58	0.10 ± 0.07	0.47 ± 0.16
500	5000	60	119 ± 40	0.09 ± 0.05	0.08 ± 0.14	173 ± 99	0.08 ± 0.04	0.58 ± 0.01
500	5000	240	203 ± 16	0.17 ± 0.19	0.59 ± 0.10	241 ± 106	0.09 ± 0.02	2.47 ± 0.54
								
2500	5000	0	1900 ± 404	0.00 ± 0.00	0.00 ± 0.00	2134 ± 403	0.00 ± 0.00	0.00 ± 0.00
2500	5000	1	955 ± 485	0.08 ± 0.09	0.95 ± 0.23	912 ± 475	0.11 ± 0.02	1.00 ± 0.29
2500	5000	10	970 ± 435	0.08 ± 0.05	0.96 ± 0.37	941 ± 501	0.08 ± 0.07	1.09 ± 0.46
2500	5000	30	1005 ± 446	0.08 ± 0.03	0.88 ± 0.27	1234 ± 551	0.07 ± 0.07	1.31 ± 0.52
2500	5000	60	1100 ± 543	0.08 ± 0.07	1.02 ± 0.01	1348 ± 511	0.12 ± 0.08	1.76 ± 0.77
2500	5000	240	1424 ± 626	0.23 ± 0.17	2.37± 0.89	1762 ± 308	0.34 ± 0.13	12.7 ± 3.2
								
5000	5000	0	3941 ± 721	0.00 ± 0.00	0.01 ± 0.02	3710 ± 698	0.00 ± 0.00	0.00 ± 0.00
5000	5000	1	2423 ± 981	0.11 ± 0.05	1.27 ± 0.80	2241 ± 1078	0.08 ± 0.03	1.27 ± 0.53
5000	5000	10	2399 ± 1152	0.08 ± 0.03	1.15 ± 0.73	2402 ± 1079	0.10 ± 0.04	1.16 ± 0.60
5000	5000	30	2509 ± 979	0.08 ± 0.02	1.07 ± 0.44	2733 ± 730	0.14 ± 0.05	2.15 ± 1.18
5000	5000	60	2550 ± 1113	0.10 ± 0.03	1.24 ± 0.37	3039 ± 791	0.16 ± 0.05	3.33 ± 1.49
5000	5000	240	3404 ± 882	0.19 ± 0.15	3.41 ± 1.74	3529 ± 489	0.65 ± 0.13	23.8 ± 5.3
								
7500	5000	0	5602 ± 1074	0.00 ± 0.00	0.00 ± 0.00	5548 ± 1059	0.00 ± 0.00	0.00 ± 0.00
7500	5000	1	3810 ± 1387	0.13 ± 0.03	1.64 ± 1.03	3800 ± 1493	0.09 ± 0.03	1.28 ± 0.51
7500	5000	10	3806 ± 1433	0.10 ± 0.03	1.50 ± 0.83	3904 ± 1592	0.15 ± 0.07	1.52 ± 0.82
7500	5000	30	3852 ± 1509	0.12 ± 0.05	1.42 ± 0.74	4362 ± 1024	0.17 ± 0.09	3.17 ± 2.21
7500	5000	60	4102 ± 1541	0.12 ± 0.05	1.47 ± 0.49	4762± 1527	0.26 ± 0.11	5.95 ± 3.17
7500	5000	240	5425 ± 1157	0.25 ± 0.15	4.48 ± 2.12	5117 ± 369	0.85 ± 0.27	29.3 ± 8.4
								
10,000	5000	0	7619 ± 1740	0.00 ± 0.00	0.00 ± 0.00	7273 ± 1046	0.00 ± 0.00	0.00 ± 0.00
10,000	5000	1	5467 ± 2057	0.10 ± 0.09	1.92 ± 1.18	5276 ± 1839	0.10 ± 0.01	1.59 ± 0.86
10,000	5000	10	5495 ± 1793	0.15 ± 0.02	1.70 ± 1.10	5740 ± 1583	0.14 ± 0.04	1.61 ± 0.97
10,000	5000	30	5644 ± 1923	0.13 ± 0.03	1.35 ± 0.67	6019 ± 1552	0.23 ± 0.09	4.08 ± 2.80
10,000	5000	60	5077 ± 1582	0.14 ± 0.07	1.70 ± 0.85	6225 ± 1529	0.31 ± 0.13	8.0 ± 3.6
10,000	5000	240	7569 ± 1475	0.29 ± 0.17	7.41 ± 2.92	7179 ± 1114	1.06 ± 0.22	39.0 ± 5.4

**Table 3:** Concentration (mean ± SD; µM) of remaining Lys and formed CML and CEL from the reaction of Lys with GO and MGO at initial concentrations of 500–10,000 µM and 5000 µM each, respectively, at 37 °C at 80 °C for the indicated times (min) in 100 mM phosphate buffer (pH 7.4).

[Lys]_o_	[GO]_o_ + [MGO]	Time	[Lys]	[CML]	[CEL]	[Lys]	[CML]	[CEL]
			37 °C			80 °C		
500	5000	0	582 ± 37	0.00 ± 0.00	0.00 ± 0.00	611 ± 27	0.03 ± 0.04	0.00 ± 0.00
500	5000	1	187 ± 75	0.48 ± 0.14	0.50 ± 0.17	216 ± 16	0.46 ± 0.19	0.59 ± 0.14
500	5000	10	228 ± 55	0.49 ± 0.11	0.52 ± 0.21	156 ± 32	0.72 ± 0.05	0.72 ± 0.03
500	5000	30	200 ± 41	0.47 ± 0.17	0.62 ± 0.19	198 ± 9	1.16 ± 0.30	0.83 ± 0.25
500	5000	60	212 ± 57	0.49 ± 0.16	0.63 ± 0.15	218 ± 70	2.18 ± 0.26	1.12 ± 0.53
500	5000	240	227 ± 33	0.59 ± 0.18	0.67 ± 0.10	183 ± 65	5.31 ± 0.54	1.97 ± 0.76
								
2500	5000	0	2861 ± 99	0.02 ± 0.02	0.00 ± 0.00	2653 ± 163	0.02± 0.01	0.00 ± 0.00
2500	5000	1	1191 ± 348	3.10 ± 1.14	2.74 ± 0.87	1190 ± 314	3.44 ± 0.81	2.73 ± 0.64
2500	5000	10	1184 ± 440	4.14 ± 0.91	2.96 ± 1.08	1192 ± 308	4.30 ± 1.16	2.71 ± 0.80
2500	5000	30	1140 ± 247	3.21 ± 1.27	2.49 ± 0.83	1145 ± 312	8.91 ± 0.89	3.25 ± 0.90
2500	5000	60	1194 ± 331	3.93 ± 1.15	2.97 ± 0.70	1365 ± 338	18.1 ± 2.34	4.26 ± 1.60
2500	5000	240	1289 ± 252	3.80 ± 1.14	2.91 ± 0.71	1593 ± 321	41.0 ± 3.7	10.9 ± 4.96
								
5000	5000	0	4938 ± 305	0.01 ± 0.01	0.00 ± 0.00	4835 ± 195	0.01 ± 0.01	0.00 ± 0.00
5000	5000	1	2618 ± 992	12.9 ± 3.6	3.82 ± 1.50	2377 ± 778	10.9 ± 4.6	3.56 ± 1.09
5000	5000	10	2543 ± 923	12.0 ± 4.1	3.59 ± 1.56	2593 ± 636	13.9 ± 2.0	3.74 ± 1.19
5000	5000	30	1915 ± 395	8.24 ± 2.63	3.56 ± 1.46	2860 ± 569	28.7 ± 3.2	4.67 ± 1.98
5000	5000	60	2351 ± 893	12.0 ± 1.1	3.61 ± 1.39	3026 ± 612	53.2 ± 0.9	7.12 ± 3.50
5000	5000	240	2747 ± 530	11.7 ± 4.3	3.85 ± 1.19	3180 ± 393	93.4 ± 10.9	19.7 ± 8.17
								
7500	5000	0	7346 ± 287	0.02 ± 0.02	0.00 ± 0.00	7111 ± 271	0.02 ± 0.02	0.00 ± 0.00
7500	5000	1	4240 ± 1156	21.8 ± 7.0	3.60 ± 1.41	3979 ± 1149	20.8 ± 7.8	3.29 ± 1.49
7500	5000	10	4098 ± 1094	21.1 ± 7.0	3.38 ± 1.26	4041 ± 717	25.7 ± 3.8	3.82 ± 1.71
7500	5000	30	4075 ± 1192	22.0 ± 6.9	3.77 ± 1.65	4481 ± 619	52.0 ± 3.6	6.02 ± 3.03
7500	5000	60	4032 ± 1135	21.9 ± 6.0	3.60 ± 2.13	4701± 739	91.8 ± 6.3	10.8 ± 5.5
7500	5000	240	4388 ± 684	24.2 ± 8.1	4.67 ± 1.67	4881 ± 649	147 ± 20	26.9 ± 9.9
								
10,000	5000	0	9884 ± 601	0.04 ± 0.01	0.00 ± 0.00	8671 ± 1218	0.02 ± 0.02	0.00 ± 0.00
10,000	5000	1	6014 ± 1498	32.4 ± 10.4	3.38 ± 1.46	5799 ± 1460	31 ± 11	3.19 ± 1.45
10,000	5000	10	5221 ± 1419	31.1 ± 6.2	3.62 ± 1.72	6173 ± 822	40 ± 6	4.05 ± 1.81
10,000	5000	30	5745 ± 1119	30.8 ± 10.6	3.24 ± 1.58	5681 ± 660	75 ± 7	8.24 ± 4.85
10,000	5000	60	5502 ± 1455	31.2 ± 9.6	3.81 ± 2.38	6311 ± 762	132 ± 2	14.8 ± 8.2
10,000	5000	240	5898 ± 481	36.6 ± 11.1	6.52± 2.33	6560 ± 703	200 ± 37	30.8 ± 10.2

**Table 4:** Concentration (mean ± SD; µM) of remaining Lys and formed CML and CEL from the reaction of Lys and GO at initial concentrations of 5000 µM and 500–10,000 µM, respectively, at 37 °C and at 80 °C for the indicated times (min) in 100 mM phosphate buffer (pH 7.4), and molar ratios of CML and CEL. n.d., not detected; n.a., not applicable.

[GO]_o_	Time	[Lys]	[CML]	[CEL]	[Lys]	[CML]	[CEL]	[CML]/[CEL]	[CML]/CEL
		37 °C			80 °C			37 °C	80 °C
500	0	3810 ± 346	0.00 ± 0.00	n.d.	3690 ± 459	0.01 ± 0.01	n.d.	0	0.1
500	1	3485 ± 81	1.46 ± 0.69	n.d.	3658 ± 527	1.96 ± 0.16	n.d.	178	16
500	10	3745 ± 496	1.52 ± 0.78	n.d.	3568 ± 434	1.72 ± 0.89	n.d.	184	21
500	30	3787 ± 365	1.95 ± 0.42	n.d.	3557 ± 334	1.99 ± 0.83	n.d.	157	24
500	60	3748 ± 443	1.85 ± 0.96	n.d.	3560 ± 463	2.96 ± 1.24	n.d.	224	36
500	240	4576 ± 69	2.09 ± 0.94	n.d.	4360 ± 241	7.43 ± 0.82	n.d.	n.a.	n.a.
									
2500	0	3521 ± 95	0.00 ± 0.00	n.d.	3948 ± 420	0.00 ± 0.00	n.d.	0	0.1
2500	1	3788 ± 987	12.2 ± 4.4	n.d.	2793 ± 27	8.8 ± 4.0	n.d.	1473	108
2500	10	3098 ± 168	11.1 ± 1.34	n.d.	3089 ± 322	9.5 ± 4.0	n.d.	1339	117
2500	30	3318 ± 303	11.3 ± 3.46	n.d.	3164 ± 425	12.5 ± 4.5	n.d.	909	154
2500	60	3239 ± 37	10.7 ± 3.1	n.d.	3186 ± 424	16.5 ± 6.7	n.d.	865	202
2500	240	4101 ± 61	17.3 ± 0.94	n.d.	4265 ± 251	50.6 ± 4.7	n.d.	n.a.	n.a.
									
5000	0	4509 ± 332	0.03 ± 0.06	n.d.	4287 ± 341	0.02 ± 0.03	n.d.	n.a.	n.a.
5000	1	3226 ± 518	22.6 ± 6.8	n.d.	3078 ± 698	20.1 ± 7.9	n.d.	n.a.	n.a.
5000	10	3284 ± 570	21.5 ± 5.6	n.d.	3276 ± 622	21.1 ± 9.0	n.d.	n.a.	n.a.
5000	30	3353 ± 401	22.2 ± 7.1	n.d.	3706 ± 272	36.6 ± 4.3	n.d.	n.a.	n.a.
5000	60	3648 ± 356	21.9 ± 3.2	n.d.	3920 ± 176	36.0 ± 3.3	n.d.	n.a.	n.a.
5000	240	4338 ± 742	22.7 ± 9.6	n.d.	4059 ± 245	107 ± 14	n.d.	n.a.	n.a.
									
7500	0	3838 ± 28	0.00 ± 0.00	n.d.	3970 ± 432	0.01 ± 0.02	n.d.	0	0.4
7500	1	2652 ± 268	15.8 ± 5.1	n.d.	2765 ± 349	17.4 ± 1.9	n.d.	898	142
7500	10	2710 ± 227	15.3 ± 4.0	n.d.	2535 ± 378	17.6 ± 3.8	n.d.	437	215
7500	30	2660 ± 420	16.6 ± 4.7	n.d.	2777 ± 328	32.6 ± 0.1	n.d.	504	266
7500	60	2698 ± 311	17.4 ± 5.1	n.d.	2794 ± 281	57.5 ± 4.8	n.d.	1329	469
7500	240	3634 ± 154	36.2 ± 2.7	n.d.	3892 ± 152	197 ± 14	n.d.	n.a.	n.a.
									
10,000	0	4007 ± 469	0.01 ± 0.02	n.d.	4238 ± 384	0.04 ± 0.07	n.d.	3.2	1.5
10,000	1	2642 ± 279	17.9 ± 2.3	n.d.	2472 ± 393	16.2 ± 3.9	n.d.	618	199
10,000	10	2472 ± 293	16.3 ± 3.1	n.d.	2642 ± 304	21.3 ± 4.2	n.d.	912	173
10,000	30	2775 ± 308	19.1 ± 3.7	n.d.	2648 ± 581	34.6 ± 11.4	n.d.	756	424
10,000	60	2530 ± 319	17.6 ± 5.3	n.d.	2619 ± 277	77.5 ± 6.6	n.d.	543	632
10,000	240	3577 ± 134	37.1 ± 3.3	n.d.	3773 ± 56	294 ± 32	n.d.	n.a.	n.a.

**Table 5:** Concentration (mean ± SD; µM) of remaining Lys and formed CML and CEL from the reaction of Lys and MGO at initial concentrations of 5000 µM and 500–10,000 µM, respectively, at 37 °C and at 80 °C for the indicated times (min) in 100 mM phosphate buffer (pH 7.4), and molar ratios of CML and CEL. n.a., not applicable.

[MGO]	Time	[Lys]	[CML]	[CEL]	[Lys]	[CML]	[CEL]	[CML]/[CEL]	[CML]/[CEL]
		37 °C			80 °C			37 °C	80 °C
500	0	3849 ± 305	0.01± 0.01	0.00 ± 0.00	3643 ± 376	0.00 ± 0.00	0.00 ± 0.00	0.1	0.0
500	1	3823 ± 297	0.06 ± 0.01	0.59 ± 0.28	3476 ± 371	0.05 ± 0.02	0.49 ± 0.13	0.1	0.1
500	10	3646 ± 407	0.04 ± 0.03	0.43 ± 0.22	3440 ± 411	0.04 ± 0.03	0.50 ± 0.10	0.1	0.1
500	30	3737 ± 444	0.03 ± 0.04	0.43 ± 0.20	3429 ± 296	0.05 ± 0.02	0.46 ± 0.19	0.1	0.1
500	60	3592 ± 319	0.06 ± 0.02	0.40 ± 0.21	3464 ± 390	0.06 ± 0.01	0.44 ± 0.16	0.2	0.1
500	240	3577 ± 248	0.09 ± 0.01	0.87 ± 0.14	4366 ± 182	0.10 ± 0.04	2.41 ± 0.30	0.1	0.1
									
2500	0	3537 ± 549	0.01± 0.01	0.00 ± 0.00	3405 ± 712	0.00 ± 0.00	0.00 ± 0.00	0.1	n.a.
2500	1	2927 ± 472	0.09 ± 0.04	0.95 ± 0.25	3006 ± 313	0.13 ± 0.01	1.26 ± 0.24	0.1	0.1
2500	10	2794 ± 496	0.11 ± 0.06	0.86 ± 0.22	2811 ± 501	0.09 ± 0.03	1.08 ± 0.39	0.1	0.1
2500	30	2734 ± 609	0.09 ± 0.06	0.90 ± 0.43	2925 ± 588	0.13 ± 0.04	1.11 ± 0.49	0.1	0.1
2500	60	2822 ± 568	0.10 ± 0.05	0.90 ± 0.33	2852 ± 550	0.11 ± 0.03	1.57 ± 0.76	0.1	0.1
2500	240	3790 ± 273	0.19 ± 0.04	2.59 ± 0.48	3967 ± 104	0.33 ± 0.03	10.6 ± 1.5	0.1	0.1
									
5000	0	3941 ± 721	0.00± 0.00	0.01 ± 0.02	3710 ± 698	0.00 ± 0.00	0.00 ± 0.00	0.0	0.0
5000	1	2423 ± 981	0.11 ± 0.05	1.27 ± 0.80	2241 ± 1078	0.08 ± 0.03	1.26 ± 0.53	0.1	0.1
5000	10	2399 ± 1153	0.08 ± 0.03	1.15 ± 0.73	2402 ± 1079	0.10 ± 0.04	1.16 ± 0.60	0.1	0.1
5000	30	2509 ± 979	0.08 ± 0.02	1.07 ± 0.44	2733 ± 750	0.14 ± 0.05	2.15 ± 1.18	0.1	0.1
5000	60	2550 ± 1113	0.10 ± 0.03	1.24 ± 0.37	3039 ± 791	0.16 ± 0.05	3.33 ± 1.49	0.2	0.2
5000	240	3404 ± 882	0.19 ± 0.15	3.41 ± 1.74	3529 ± 489	0.65 ± 0.13	23.8 ± 5.3	0.1	0.1
									
7500	0	3606 ± 709	0.00± 0.00	0.00 ± 0.00	3645 ± 625	0.00 ± 0.00	0.00 ± 0.00	0.1	n.a.
7500	1	1752 ± 428	0.14 ± 0.06	1.33 ± 0.59	1837 ± 510	0.11 ± 0.05	1.26 ± 0.35	0.1	0.1
7500	10	1872 ± 577	0.14 ± 0.07	1.33 ± 0.49	1821 ± 253	0.15 ± 0.06	1.38 ± 0.59	0.1	0.1
7500	30	1957 ± 399	0.13 ± 0.05	1.28 ± 0.41	2321 ± 560	0.19 ± 0.09	2.38 ± 1.10	0.1	0.1
7500	60	2001 ± 505	0.13 ± 0.05	1.24 ± 0.33	2291 ± 338	0.23 ± 0.13	3.65 ± 1.78	0.1	0.1
7500	240	2437 ± 359	0.34 ± 0.10	4.66 ± 0.86	3484 ± 91	0.69 ± 0.06	26.5 ± 3.7	0.1	0.1
									
10,000	0	3690 ± 638	0.00± 0.00	0.00 ± 0.00	3822 ± 758	0.00 ± 0.00	0.00 ± 0.00	0.1	n.a.
10,000	1	1447 ± 429	0.12 ± 0.04	1.48 ± 0.42	1386 ± 496	0.13 ± 0.06	1.65 ± 0.81	0.1	0.1
10,000	10	1390 ± 326	0.13 ± 0.06	1.29 ± 0.51	1582 ± 385	0.16 ± 0.09	1.60 ± 0.50	0.1	0.1
10,000	30	1428 ± 262	0.17 ± 0.10	1.62 ± 0.57	1955 ± 477	0.23 ± 0.12	2.91 ± 1.47	0.1	0.1
10,000	60	1672 ± 591	0.14 ± 0.06	1.61 ± 0.76	2610 ± 100	0.34 ± 0.05	6.64 ± 0.1	0.1	0.1
10,000	240	2065 ± 250	0.36 ± 0.06	5.04 ± 0.12	3008± 147	0.77 ± 0.10	30.0 ± 3.8	0.1	0.1

**Table 6:** Concentration (mean ± SD; µM) of remaining Lys and formed CML and CEL from the reaction of Lys (5000 µM) with GO (5000 µM) and MGO (500–10,000 µM), respectively, at 37 °C and at 80 °C for the indicated times (min) in 100 mM phosphate buffer (pH 7.4), and molar ratios of CML and CEL. n.a., not applicable.

[GO]_o_	[MGO]_o_	Time	[Lys]	[CML]	[CEL]	[Lys]	[CML]	[CEL]	[CML]/[CEL]	[CML]/[CEL]
			37 °C			80 °C			37 °C	80 °C
5000	500	0	3587 ± 632	0.00 ± 0.00	0.01 ± 0.02	3657 ± 774	0.00 ± 0.00	0.00 ± 0.00	0	n.a.
5000	500	1	2370 ± 844	12.3 ± 6.4	0.00 ± 0.00	2378 ± 561	11.5 ± 2.3	0.04 ± 0.06	n.a.	108
5000	500	10	2524 ± 663	12.2 ± 4.7	0.07 ± 0.12	2430 ± 489	10.3 ± 4.0	0.12 ± 0.11	60	57
5000	500	30	2489 ± 673	12.3 ± 4.4	0.00 ± 0.00	2387 ± 560	17.9 ± 5.0	0.15 ± 0.19	n.a	79
5000	500	60	2462 ± 462	11.8 ± 4.4	0.05 ± 0.09	2441 ± 440	27.2 ± 9.6	0.28 ± 0.25	79	65
5000	500	240	3703 ± 142	24.8 ± 4.4	0.39 ± 0.14	4085 ± 80	113 ± 9	2.5 ± 0.8	64	45
										
5000	2500	0	3707 ± 552	0.00 ± 0.00	0.00 ± 0.00	3642 ± 546	0.01 ± 0.02	0.00 ± 0.00	n.a.	n.a.
5000	2500	1	2068 ± 596	6.7 ± 2.2	0.65 ± 0.22	1977 ± 730	6.28 ± 2.5	0.79 ± 0.45	10	8
5000	2500	10	2318 ± 584	7.6 ± 2.3	0.56 ± 0.42	2073 ± 587	7.1 ± 2.4	0.67 ± 0.28	14	11
5000	2500	30	2163 ± 642	7.9 ± 2.9	0.66 ± 0.28	2192 ± 473	14.4 ± 4.7	0.71 ± 0.30	12	20
5000	2500	60	2303 ± 397	7.6 ± 2.6	0.57 ± 0.23	2333 ± 428	26.1 ± 9.2	1.21 ± 0.57	13	22
5000	2500	240	3045 ± 478	19.3 ± 1.9	2.19 ± 0.85	3867 ± 33	120 ± 13	9.6 ± 2.7	9	13
										
5000	5000	0	4938 ± 305	0.01 ± 0.01	0.00 ± 0.00	4835 ± 195	0.01 ± 0.01	0.00 ± 0.00	1	0.1
5000	5000	1	2618 ± 992	12.9 ± 3.6	3.82 ± 1.50	2377 ± 778	10.8 ± 4.6	3.6 ± 1.1	3	3
5000	5000	10	2543 ± 923	12.0 ± 4.1	3.59 ± 1.56	2593 ± 636	13.9 ± 2.0	3.7 ± 1.1	3	4
5000	5000	30	1915 ± 395	13.8 ± 9.7	3.56 ± 1.46	2860 ± 569	28.7 ± 3.2	4.7 ± 2.0	4	6
5000	5000	60	2351 ± 893	12.0 ± 1.1	3.61 ± 1.39	3026 ± 612	53 ± 1	7.1 ± 3.5	4	7
5000	5000	240	2747 ± 530	11.7 ± 4.3	3.85 ± 1.19	3180 ± 393	93 ± 11	20 ± 8	3	5
										
5000	7500	0	4996 ± 557	0.00 ± 0.00	0.00 ± 0.00	5251 ± 274	0.01 ± 0.01	0.00 ± 0.00	n.a.	n.a.
5000	7500	1	2044 ± 649	8.4 ± 1.9	4.03 ± 0.82	2023 ± 234	9.8 ± 1.7	3.9 ± 0.9	2	3
5000	7500	10	2006 ± 606	9.6 ± 0.5	4.20 ± 1.45	1922 ± 278	9.2 ± 1.8	3.8 ± 0.4	2	2
5000	7500	30	1873 ± 744	8.7 ± 1.9	4.26 ± 0.56	2234 ± 262	22.1 ± 6.7	4.9 ± 1.0	2	5
5000	7500	60	1622 ± 130	11.1 ± 0.6	4.51 ± 0.59	2972 ± 614	41.8 ± 10	8.0 ± 2.5	2	5
5000	7500	240	2054 ± 498	10.0 ± 1.8	5.03 ± 0.72	3158 ± 95	102 ± 6	22.1 ± 4.6	2	5
										
5000	10,000	0	5482 ± 122	0.01 ± 0.01	0.00 ± 0.00	5313 ± 236	0.01 ± 0.01	0.00 ± 0.00	n.a.	n.a.
5000	10,000	1	1886 ± 334	5.5 ± 0.5	4.06 ± 0.40	1659 ± 183	7.7 ± 1.5	4.3 ± 0.7	1	2
5000	10,000	10	1944 ± 506	7.4 ± 3.7	4.30 ± 0.96	1598 ± 340	9.1 ± 1.4	4.6 ± 0.6	2	2
5000	10,000	30	1796 ± 346	5.4 ± 1.2	4.18 ± 0.30	1968 ± 166	20.1 ± 6.3	6.1 ± 1.2	1	3
5000	10,000	60	1854 ± 308	6.6 ± 0.5	4.52 ± 0.96	2453 ± 226	43.6 ± 10.4	9.8 ± 3.3	1	4
5000	10,000	240	2172 ± 512	8.3 ± 2.5	5.22 ± 0.78	2862 ± 143	95 ± 10	27.4 ± 5.0	2	3

**Table 7:** Concentration (mean ± SD; µM) of remaining Lys and formed CML and CEL from the reaction of Lys (5000 µM) with GO (500, 2500, 7500, 10,000 µM) and MGO (10,000 µM), respectively, at 37 °C and at 80 °C for the indicated times (min) in 100 mM phosphate buffer (pH 7.4), and molar ratios of CML and CEL. n.a., not applicable.

[GO]_o_	[MGO]_o_	Time	[Lys]	[CML]	[CEL]	[Lys]	[CML]	[CEL]	[CML]/[CEL]	[CML]/[CEL]
			37 °C			80 °C			37 °C	80 °C
500	5000	0	4796 ± 154	0.00 ± 0.00	0.00 ± 0.00	4856 ± 60	0.00 ± 0.00	0.00 ± 0.00	n.a.	n.a.
500	5000	1	3159 ± 359	2.27 ± 0.16	2.88 ± 0.98	3112 ± 261	2.2 ± 0.2	2.7 ± 0.5	1	1
500	5000	10	3193 ± 389	2.27 ± 0.09	2.73 ± 0.70	3270 ± 465	2.5 ± 0.3	2.7 ± 0.4	1	1
500	5000	30	3045 ± 498	2.20 ± 0.19	2.72 ± 0.84	3555 ± 17	3.7 ± 0.3	3.7 ± 0.9	1	1
500	5000	60	2995 ± 609	2.16 ± 0.18	2.69 ± 1.02	3731 ± 114	5.3 ± 0.5	6.4 ± 1.4	1	1
500	5000	240	3088 ± 491	2.18 ± 0.28	3.17 ± 0.63	3669 ± 87	7.3 ± 1.0	18.2 ± 3.4	1	1
										
2500	5000	0	4965 ± 110	0.00 ± 0.00	0.00 ± 0.00	4901 ± 208	0.01 ± 0.01	0.00 ± 0.00	n.a.	n.a.
2500	5000	1	2654 ± 197	8.3 ± 1.0	2.76 ± 0.59	2753 ± 289	8.2 ± 0.9	2.6 ± 0.5	3	8
2500	5000	10	2887 ± 510	8.4 ± 1.1	2.69 ± 0.54	2908 ± 273	8.8 ± 0.2	2.6 ± 0.2	3	11
2500	5000	30	2807 ± 252	8.6 ± 0.7	2.78 ± 0.34	3242 ± 264	17.4 ± 3.0	3.2 ± 0.6	3	20
2500	5000	60	2826 ± 352	8.2 ± 0.7	2.82 ± 0.58	3493 ± 333	29.3 ± 3.8	6.0 ± 1.4	3	22
2500	5000	240	2778 ± 405	9.1 ± 0.5	3.38 ± 0.78	3537 ± 140	44.2 ± 4.1	16.8 ± 3.4	3	13
										
7500	5000	0	5257 ± 149	0.28 ± 0.49	0.00 ± 0.00	5010 ± 163	0.00 ± 0.00	0.00 ± 0.00	n.a.	n.a.
7500	5000	1	2899 ± 224	15.1 ± 3.1	2.67 ± 0.47	2549 ± 303	14.1 ± 0.9	2.9 ± 0.6	6	5
7500	5000	10	2764 ± 41	13.5 ± 0.9	2.84 ± 0.38	2670 ± 485	17.8 ± 2.7	2.7 ± 0.2	5	7
7500	5000	30	2820 ± 103	14.1 ± 0.9	2.83 ± 0.24	2917 ± 243	40.2 ± 7.3	3.5 ± 0.6	5	11
7500	5000	60	2833 ± 120	14.7 ± 1.3	2.78 ± 0.36	3121 ± 133	86 ± 15	5.2 ± 0.7	5	16
7500	5000	240	2749 ± 512	15.4 ± 1.6	3.10 ± 0.25	3333 ± 172	181 ± 13	16.0 ± 4.5	5	11
										
10,000	5000	0	4888 ± 805	0.00 ± 0.00	0.00 ± 0.00	5135 ± 493	0.03 ± 0.03	0.00 ± 0.00	n.a.	n.a.
10,000	5000	1	2650 ± 112	16.1 ± 2.3	3.01 ± 0.28	2604 ± 141	15.8 ± 1.0	3.1 ± 0.4	5	5
10,000	5000	10	2574 ± 314	14.7 ± 0.8	2.97 ± 0.31	2754 ± 271	20.7 ± 2.5	3.3 ± 0.3	5	6
10,000	5000	30	2680 ± 345	16.6 ± 1.2	2.93 ± 0.49	2737 ± 274	49.7 ± 12.7	3.7 ± 0.8	6	13
10,000	5000	60	2710 ± 465	16.1 ± 1.2	2.93 ± 0.42	2842 ± 84	106 ± 23	5.0 ± 1.5	6	21
10,000	5000	240	2750 ± 297	18.6 ± 0.8	3.16 ± 0.45	2998 ± 117	255 ± 21	13.6 ± 3.4	6	19

## Data Availability

Not Applicable.

## Supplementary Materials

The following supporting information can be downloaded at: https://www.mdpi.com/article/10.3390/ijms23073446/s1.
