# Supplementary material for: Free L-Lysine and Its Methyl Ester React with Glyoxal and Methylglyoxal in Phosphate Buffer (100 mM, pH 7.4) to Form Nε-Carboxymethyl-Lysine, Nε-Carboxyethyl-Lysine and Nε-Hydroxymethyl-Lysine
Source: Int J Mol Sci. 2022 Mar 22;23(7):3446. doi: 10.3390/ijms23073446 (PMC8998464; doi:10.3390/ijms23073446)
Supplement: Supplementary file 1 [file ijms-23-03446-s001.zip › ijms-1622526-supplementary.pdf]

Supplement to:

Free L-lysine and its methyl ester react with glyoxal and methylglyoxal in phosphate buffer (100 mM, pH 7.4) to form N^{ε}-carboxymethyl-lysine, N^{ε}-carboxyethyl-lysine and N^{ε}-hydroxymethyl-lysine

Svetlana Baskal, Dimitrios Tsikas,*

[1]Institute of Toxicology, Core Unit Proteomics, Hannover Medical School, Hannover, Germany

After addition of the chemicals to the buffer, the maximum wavelengths were 208 nm for Lys in the absence of MGO and 273 nm for MGO in the absence of Lys (Fig. S1). The UV/vis spectra of mixtures containing both Lys and MGO differed from those of Lys and MGO alone and dependent upon the order of their addition to the buffer. Higher absorbance values were obtained when first Lys and then MGO was added Fig. S1. The UV/vis spectra of the reaction mixtures show Lys-concentration-depending absorbance values, which were higher in the presence of MGO and GO+MGO compared to GO (Fig. S2). In the presence of GO and MGO, GO only marginally reduced the absorbance values caused Lys and MGO. The absorbance at 273 nm of the sample that contained MGO but Lys only little changed with reaction time. The absorbance at 273 nm of the sample that contained MGO and Lys increase more strongly with reaction time (Fig. S3).

The reaction mixtures of Lys and GO or Lys and MGO were almost colourless at 37 °C at all concentrations tested. At 80 °C, the reaction mixtures of Lys and GO were slightly yellowish only at high concentrations. At this temperature, the reaction mixtures of Lys and MGO were yellow at lower and brownish at high concentrations.

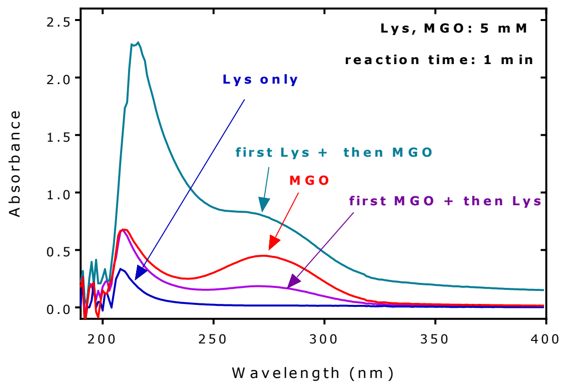

Figure S1. UV/vis spectra of buffered solutions of Lys, MGO and their mixture one minute after their addition at room temperature (about 19 °C). The concentrations were each 5 mM.

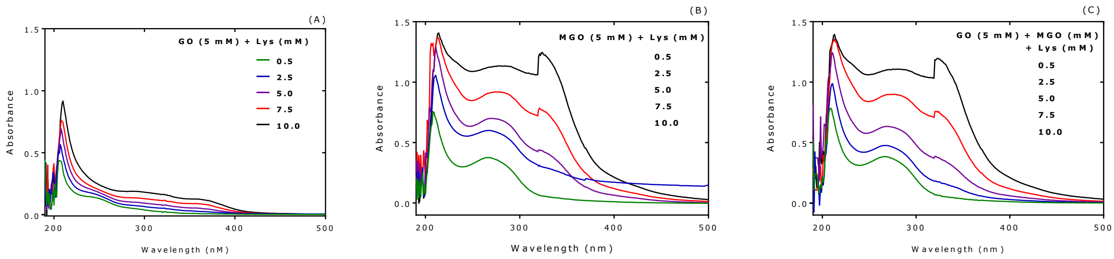

Figure S2. UV/vis spectra of buffered solutions of Lys, GO, MGO and their mixtures one minute after their addition at room temperature (about 19 °C). Lys was first added to the buffer followed by the addition of GO, MGO and the simultaneous addition of GO and MGO. The initial concentrations were 0.5, 2.5, 5, 7.5 and 10 mM for Lys, and each 5 mM for GO and MGO.

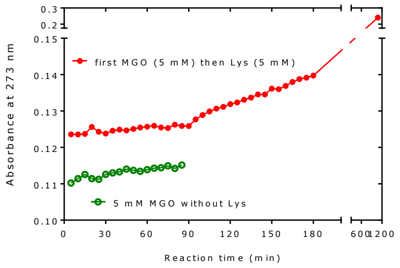

Figure S3. Time course of the absorbance at 273 nm in buffered solutions of MGO (5 mM) alone and in the presence of Lys (5 mM) (about 19 °C). Scans were performed every 5 min and at the end of the experiment (about 21 h).

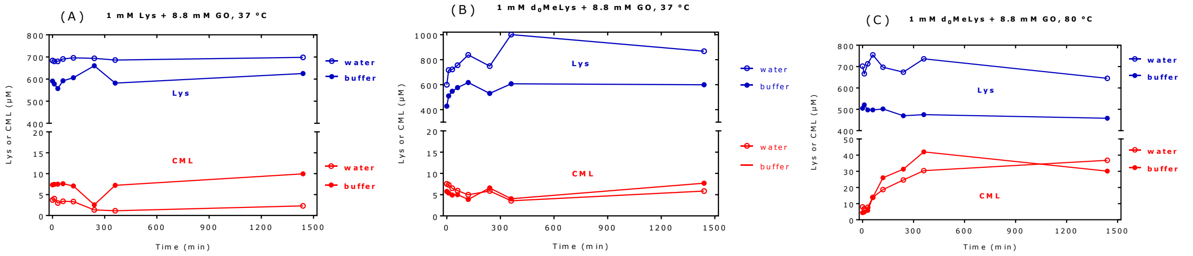

Figure S4. Time-dependent formation of CML in reaction mixtures of free Lys (1 mM) or Lys methyl ester (1 mM) GO alone (8.8 mM) in distilled water or 100 mM phosphate buffer, pH 7.4, incubated at 37 °C (A and B) or 80 °C (C) for the indicated times. All experiments were performed in three independent experiments. For the sake simplicity, only the mean values are plotted.
